# Low Energy Atomic Models Suggesting a Pilus Structure that could Account for Electrical Conductivity of *Geobacter sulfurreducens* Pili

**DOI:** 10.1038/srep23385

**Published:** 2016-03-22

**Authors:** Ke Xiao, Nikhil S. Malvankar, Chuanjun Shu, Eric Martz, Derek R. Lovley, Xiao Sun

**Affiliations:** 1 State Key Laboratory of Bioelectronics, School of Biological Science and Medical Engineering, Southeast University, Nanjing, 210096, China; 2 Department of Microbiology, University of Massachusetts, Amherst, Massachusetts, USA

## Abstract

The metallic-like electrical conductivity of *Geobacter sulfurreducens* pili has been documented with multiple lines of experimental evidence, but there is only a rudimentary understanding of the structural features which contribute to this novel mode of biological electron transport. In order to determine if it was feasible for the pilin monomers of *G. sulfurreducens* to assemble into a conductive filament, theoretical energy-minimized models of *Geobacter* pili were constructed with a previously described approach, in which pilin monomers are assembled using randomized structural parameters and distance constraints. The lowest energy models from a specific group of predicted structures lacked a central channel, in contrast to previously existing pili models. In half of the no-channel models the three N-terminal aromatic residues of the pilin monomer are arranged in a potentially electrically conductive geometry, sufficiently close to account for the experimentally observed metallic like conductivity of the pili that has been attributed to overlapping pi-pi orbitals of aromatic amino acids. These atomic resolution models capable of explaining the observed conductive properties of *Geobacter* pili are a valuable tool to guide further investigation of the metallic-like conductivity of the pili, their role in biogeochemical cycling, and applications in bioenergy and bioelectronics.

Understanding the mechanisms for electron transport along the electrically conductive Type IV pili of *Geobacter* species is important because these ‘microbial nanowires’ enable extracellular electron exchange that is of environmental and practical significance^1^,^2^,^3^,^4^,^5^,^6^. One hypothesis for the mechanism of electron transport is that electrons move along the pilus by multistep hopping between discrete carriers such as clustered or dimerized aromatic amino acids^7^,^8^. An alternative proposed mechanism is that electron transport is facilitated by overlapping pi-pi orbitals of aromatic amino acids which confer a metallic-like conductivity similar to that found in synthetic organic metallic nanostructures comprised of polyaniline or polyacetylene^9^,^10^,^11^. Experimental evidence consistent with metallic-like conductivity includes: (1) temperature and pH responses consistent with metallic-like conductivity^3^; (2) charge propagation along pili similar to that of carbon nanotubes^12^; (3) structural evidence for pi-pi stacking of aromatic amino acids^13^; and (4) a loss of pilus conductivity when key aromatic acids are genetically removed from PilA, the pilin monomer^14^.

Metallic-like conductivity has not been reported for other biological proteins. Thus a major question is how pi-pi stacking of aromatic amino acids might be achieved in *G. sulfurreducens* pili. In the absence of full structural data, homology modeling is one approach to investigate this question^7^,^8^,^13^,^15^,^16^. When the structure of *G. sulfurreducens* PilA was superimposed on a template of the pilus structure of *Neisseria gonorrhoeae*, the resulting homology models^7^,^8^,^15^,^16^ did not explain or predict the close packing of aromatic amino acids that is apparent in X-ray diffraction data^13^. In contrast, a homology model constructed with the *Pseudomonas aeruginosa* pilus structure as the template predicted that aromatic amino acids in *G. sulfurreducens* pili are packed within 3 to 4 Å, consistent with X-ray diffraction data, as well as the impact of temperature, pH, and aromatic amino acid substitution on conductivity^13^. However, alternative modeling approaches to more fully explore the possibilities of *G. sulfurreducens* pilus structure are warranted to provide more information to facilitate further hypothesis-driven experimental investigation of the conductivity mechanisms.

Another strategy for modeling pilus structure^17^ successfully reconstructed the known structures of the type IVa pili of *Neisseria gonorrhoeae*^18^; the type IVb pili of *Vibrio cholera*^19^,^20^; and the T2SS pseudopili of *Klebsiella oxytoca*^21^ from the structure of the pilin monomer, by employing the Rosetta Symmetric Docking protocol^22^ with a combination of randomized structural parameters and distance constraints. Here we report that the application of this modeling approach suggests options, different than those derived from homology modeling, for how the *G. sulfurreducens* pilus assembles to yield the experimentally observed close-packing of aromatic amino acids.

## Methods and Materials

The models of *Geobacter sulfurreducens* pili were generated with methods previously validated by reconstructing pili of known structure (*Neisseria gonorrhoeae, Vibrio cholerae*, and *Klebsiella oxytoca*)^17^. The modeling used standard symmetric docking protocols in the Rosetta software suite^22^,^23^, combined with structural parameters such as symmetry details and distance constraints.

The structure of the type IVa major pilin subunit PilA from *Geobacter sulfurreducens* was previously determined with solution state NMR spectroscopy^8^ (Protein Data Bank entry 2M7G). Conformer number one^8^, having the best clashscore and MolProbity^24^ score from this 18-model ensemble, was taken as the initial subunit model. The mature pilin subunit contains 61 amino acid residues, including 6 aromatic residues (Phe1, Phe24, Tyr27, Tyr32, Phe51 and Tyr57). The initial helical symmetry parameters (rotation angle between monomers, rise along the fiber axis between monomers, and radius from the fiber axis to the center of mass of each monomer) were weighted towards those of the GC pilus structure of *Neisseria gonorrhoeae*^18^, which is the only empirical full atomic structure of type IV pilus in the Protein Data Bank (2HIL).

**Figure 1:**
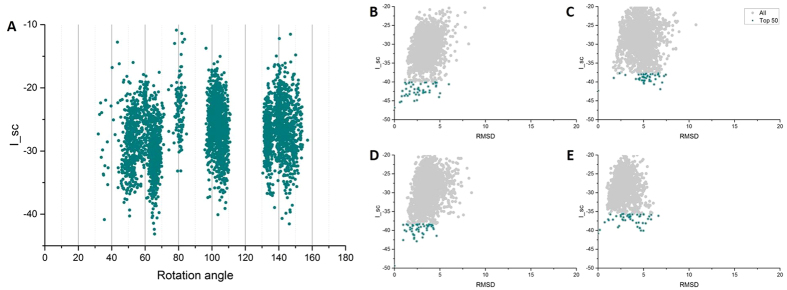
The landscapes of interfacial energy scores (I_sc). The landscapes of interfacial energy versus rotation angle of subunits at the end of the first step (**A**) show that models tend to converge into 4 local troughs which have rotation angles of 40-60°, 60-80°, 100-120°, and 130-160°. These four low-energy regions were taken as the starting range for the second step. After all the 3 steps of the modeling, the interfacial energy versus RMSD of the 40-60° group (**B**) and the 100-120° group (**D**) show good convergence towards the lowest-energy model and relatively lower energy scores, and most low-energy models from these two groups have RMSD less than 5 Å from the lowest one. The 60-80° group (**C**) and the 130-160° group (**E**) are scattered more randomly. The selected top 50 models from each group are in dark cyan.

A multi-step procedure was taken: in the first step, pilus models were assembled using the initial subunit and randomized assembly parameters. Each symmetry parameter was randomized in a Gaussian distribution. The rotation angle of subunits around the helical axis was varied from 0 to 180 degrees around a median of 90 degrees (GC pilus: 100.8 degrees). The rise per subunit was varied from 5–15 Å around a median of 10 Å (GC pilus: 10.5 Å). The radius from the subunit center of mass to the helical axis was varied from 15–30 Å around a median of 22.5 Å (GC pilus: 17.8 Å). In each initial assembly, the orientation of the subunits was varied by random rotations around the center of mass of the subunit (−20 to 20 degrees). The initial assemblies were subjected to a series of perturbations, preserving those that reduced the energy. Subunits were treated as rigid bodies in this step, and all subunits in each assembly were rotated identically. This initial assembly step converged to a small number of low-energy clusters of models (see Fig. 1A).

In the second step, for each local trough in the energy landscape, a fixed-backbone docking calculation in a narrower range of symmetry parameters was performed near the minima obtained in step one, followed by a clustering process in which models were rotated around and shifted along their symmetric axes so that the lowest Root Mean Square Differences (RMSDs) could be determined^17^,^25^, based on the Cα positions and with a cut-off of 2.00 Å. All backbone heavy atoms in a single subunit were constrained at their initial coordinates so that only the side chain torsional angles varied during this docking step. In the final step, a refinement was applied to the center of the largest cluster from each calculation in last step. Here, a series of initial perturbations were taken, with 0.7 Å for the translational perturbation and 5.0° for the rotational perturbation. And then a fast simulated annealing step was employed to relax the full atomic models, allowing flexibility of the monomer backbones while keeping the conformations of all monomers identical. More than 6,000 models were generated for the first step, and 2,000 for the second and third steps respectively. The final models were further examined by Ramachandran plot^26^ and MolProbity.

As suggested by Rosetta Symmetrical Docking protocol^22^ and the previous works about T4P modeling^17^,^21^,^25^, use of constraints can drastically reduce the conformational search space. So ambiguous distance constraints similar to previous study were utilized during the calculations. Constraints were set as: (i) a distance near 4 Å between alpha carbons for at least one pair of aromatic residues in different chains, because of the potential pi-stacking inferred from the x-ray diffraction of *G. sulfurreducens* pili^3^,^12^,^13^, and because of the distance near 4 Å between aromatic rings that might contribute to the electron transfer in conducting polymers^27^,^28^,^29^,^30^; (ii) at least one possible salt bridge between chains based on distance between oxygen and nitrogen atoms, for the reason that type IV pili and T2SS pseudopili probably use salt bridges to stabilize their structures^18^,^19^,^20^,^21^; and (iii) alpha carbons of Phe1 and Glu5 in neighboring chains should be less than 15 Å apart, as the proximity of N-terminal nitrogen and Glu5 might be conserved among most type IV pili and contribute to the assembly^31^. All the distance constraints for the method were set by a flat harmonic function^17^, and implemented by employing Rosetta Constraint Files^32^. Penalty scores were applied for each feature lacking in a model. Models with high total penalty scores were discarded. Taking aromatics as an example, the ambiguous contact was depicted by an enumeration of all the pairs of different aromatic residues from different subunits, P1, P2 … Pn. All the penalty scores of these pairs S(Pi), where i = 1, 2 … n, were calculated and then the ambiguous constraint was described by min(S(Pi)), which picked the minimum from all the scores of possible pairs. Because there are 5 aromatic residues in each subunit except for the Tyr57 on the flexible C-terminal tail, and the total number of subunits was 21 for the calculation, the ambiguous contact should be a combination of 200 possible residue pairs (4*5*10, pairs formed by the same residues from different subunits will not be counted, and only the master subunit in the middle and the upper 10 subunits are taken into account because of the symmetry).

Molecular images were prepared with PyMOL^33^ or Jmol^34^.

## Results and Discussion

### Initial Model Outputs

More than 6,000 models were generated and filtered by their diameters. The estimated diameter of *G. sulfurreducens* pili is close to 30 Å^12^,^35^,^36^, and thus only models with diameters between 30Å and 50 Å were selected for subsequent analysis. Symmetry details such as azimuthal angle, axial rise, and diameters are correlated with each other^17^. Therefore, we used the azimuthal angle as an indication of symmetry details and drew the distribution of interfacial energy, which is calculated as the difference between the total energy of the complex and the total energy when the partners are separated^37^, as a function of rotation angle, for all the filtered models. These models converged into four local troughs, which are located in 40–60°, 60–80°, 100–120° and 130–160° (Fig. 1A). These four low-energy regions were taken as the starting range for the next step.

In the second step, independent samplings were employed for each of the four low-energy regions, and four clusters of models were taken from the results of each sampling. Each cluster was selected based on Root Mean Square Deviations (RMSD) of the Cα positions, with a cut-off of 2 Å. The center of the largest cluster was chosen as the starting conformation for the next step because a native structure might be situated within a broad basin of low-energy conformations to preserve the efficiency and robustness of structure^38^.

During the third step, a local refinement was performed for each starting conformation from the end of the previous step. The refining procedure was done near the selected structures from each of the four different groups with initial rotation angles of 40–60°, 60–80°, 100–120°, and 130–160°. For each refinement process, rigid-body perturbations were followed by relaxation with flexible backbones. The 50 lowest-energy models were picked from each refinement, and the landscapes of interfacial energy versus the RMSDs from the lowest energy model are depicted in Fig. 1B–E. Two groups, 40–60° (Figs 2 and 3) and 100–120° (Fig. S2), seem more convergent and have lower interfacial energy than the other two (Figs S1 and S3). Because the 100–120° group shows similar structures to the previous models based on the *Neisseria* GC pilus^7^,^8^,^15^,^16^ which are inconsistent with experimental results^3^,^13^, all the models discussed below are from the 40–60° group.

In these models, the energy minimization process results in chemically realistic interactions between subunit chains, including shape complementarity, hydrogen bonds, salt bridges, and hydrophobic interactions. Ramachandran plots of these structures have been produced. And taking one structure as an example, the plots indicated that 96.6% of residues were in the favored region, 3.4% were in the allowed region, and no residue was in the disallowed region (Fig. S4). Also, these models have very few van der Waals clashes (about one per 1,000 atoms) as determined by the MolProbity server^24^. In contrast, a cryo-EM pilus model for the *Neisseria* pilus^18^ has 107 clashes per 1,000 atoms; and a model of the *Pseudomonas* pilus^39^ has 54 clashes per 1,000 atoms. In order to directly reflect the empirical data, those previous modeling processes did not minimize energy, and consequently lack chemically realistic interactions between subunit chains.

### A cluster of models with closely packed aromatic rings

Although different groups of low-energy models exhibit various possibilities of pili structure from the perspective of energy, one group, with an initial rotation angle between 40° and 60°, shows the close packing of aromatics demonstrated by synchrotron X-ray diffraction experiments^13^ on *G. sulfurreducens* pili. Notably, 24 of the 50 lowest energy models from this group have aromatic rings packed in a continuous chain, which was not observed in the other three groups of models. The continuous packing of aromatic rings in these models is consistent with the experimental observations suggesting that the metallic-like conductivity of the *G. sulfurreducens* pili is dependent upon close packing of aromatic amino acids.

**Figure 2:**
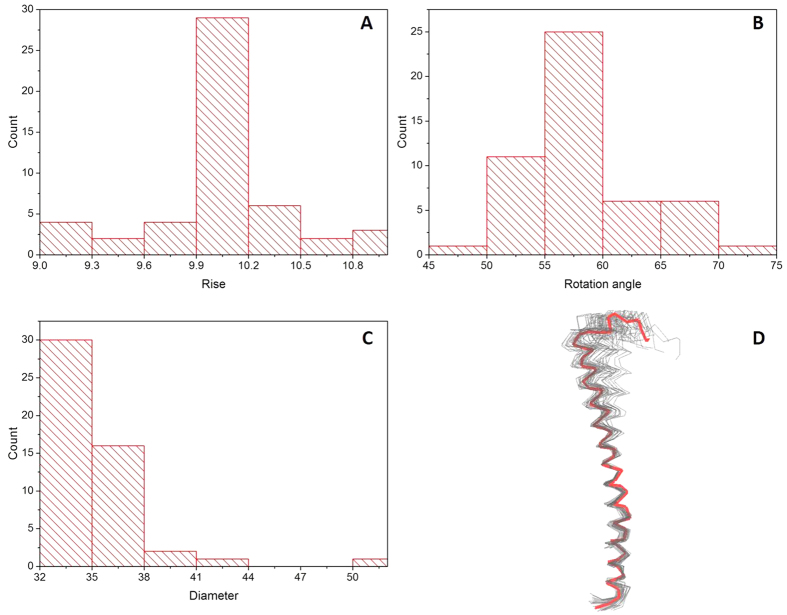
Overview of the symmetry features of the 50 lowest-energy models. (**A**) The axial rise. (**B**) The rotation angle. (**C**) The filament diameter. (**D**) Overlay of the monomers from the 50 predicted models (grey) and the experimentally determined pilin structure (red, model 1 from 2M7G).

Analysis of the 50 lowest-energy models from this group revealed that the symmetry parameters seem to center near specific values (Fig. 2), implying that these models are quite similar to each other. Most of the models have an axial rise near 10 Å; the azimuthal angles are mostly located between 55 and 60 degrees, corresponding to 6 to 6.5 subunits per turn; and the diameters of models are near 35 Å, consistent with diameter measurements^12^,^36^.

Monomers were extracted from the top 50 models, and aligned to the experimentally determined pilin structure^8^ , using residues 1–33 (Fig. 2D). The ~30 residues in the N-terminal alpha-helices, which are highly conserved in Type IVa pili^31^, show minimal deviations, but the C-termini vary somewhat more. This is consistent with the flexibility in the middle of the PilA helix^8^, which allows the filaments to bend and stretch without breaking^40^. The deviations between different monomers and the variation of helical symmetry parameters in different models suggest that a slight deformation in the monomer could influence the structure of the assembled pilus filament to some extent, which was also suggested in a molecular dynamics study of T2SS pseudopili from *Klebsiella oxytoca*^41^. Considering the fact that the pilin subunit of the *G. sulfurreducens* pili is much smaller than the subunits of other type IV pili, which will probably increase their flexibility as simulated by molecular dynamics^42^, the structure of *G. sulfurreducens* pilin is probably more flexible and variable, and may increase the difficulty of structural determination by experimental methods such as cryo-EM. Therefore, due to the flexibility and uncertain structure of the C-terminus, we have focused the discussion and interpretation in this paper on the aromatic residues in the N-terminus of the pilus.

**Figure 3:**
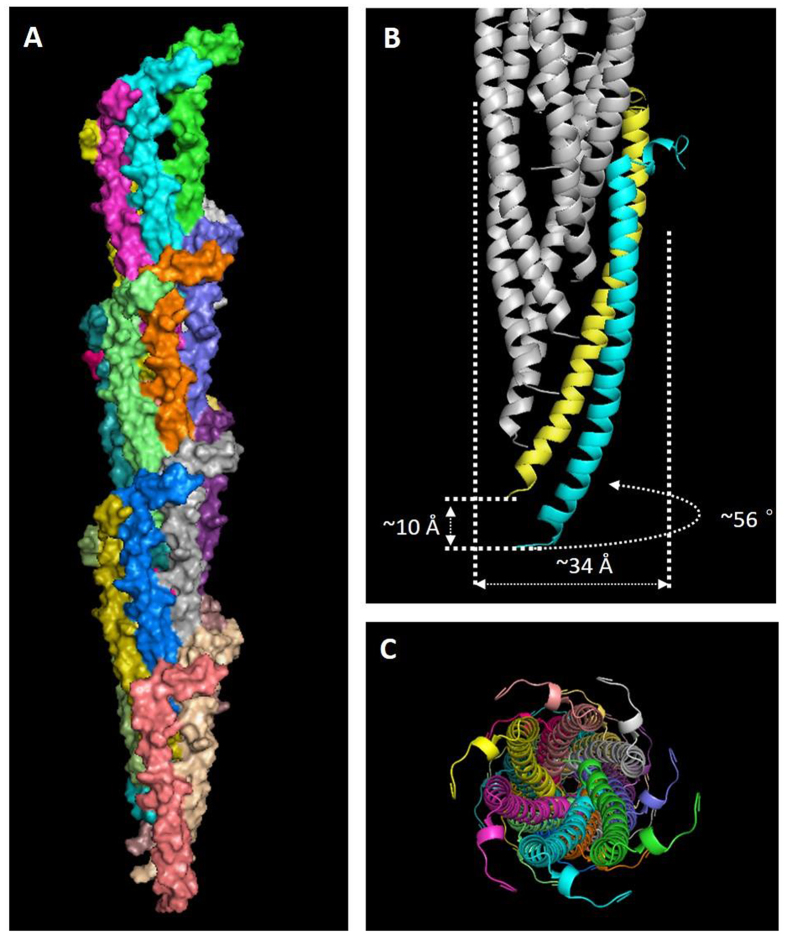
A selected structure of the *G. sulfurreducens* pilus from the 50 lowest-energy models. (**A**) The filament model containing 21 subunits, each with a different color. (**B**) The helical azimuthal angle, axial rise and diameter of the selected model. (**C**) End view of the structure.

One model with regularly aligned aromatic rings, which is most consistent with the X-ray diffraction results, was picked from the 50 models with the lowest interfacial energy scores as a representative of these structures and designated the ARC-1 (Aromatic Ring Conductivity-1) model. The subunits of ARC-1 align in a right-handed helix with a rotation angle of ~56 degrees between subunits around the helical axis (Fig. 3A–C). The translation along the axis (the axial rise) is ~10 Å, and the diameter of the filament is ~34 Å, which is consistent with previous results from atomic force microscopy^12^,^35^. The ARC-1 structure is more compact with a smaller rotation angle than the *N. gonorrhoeae* pilus, which is consistent with the fact that the *G. sulfurreducens* pilin monomer is much smaller (61 amino acids) than the *N. gonorrhoeae* pilin (158 amino acids).

### Aromatic residues may form a potentially conductive pathway

**Figure 4:**
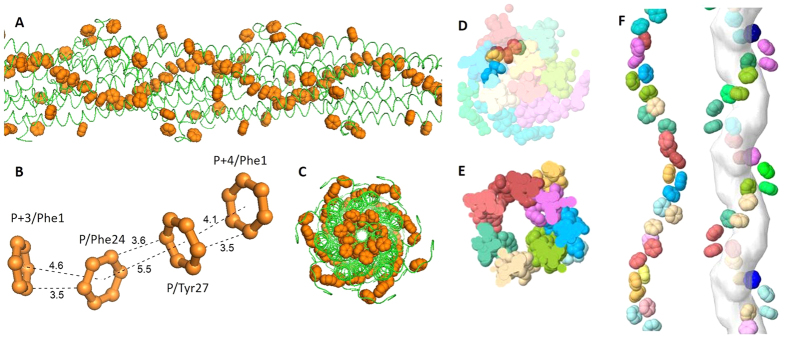
The compact chain of aromatic rings in the ARC-1 pilus model (**A**, **B**, **C** and **D**) and the comparison to Geobacter homology model based on Pseudomonas (**E**, **F**). (**A**) Overview of the aromatic rings (orange spheres) in the ARC-1 pilus model; (**B**) Details of the neighbouring aromatic rings, which repeat through the length of the filament of the ARC-1 pilus model. The closest atoms between rings are 3.5-3.6 Å apart. The centers of the rings are 4.1, 4.6, and 5.5 Å apart. “P” denotes a reference protomer. “P + 3” and “P + 4” denote 3rd and 4th protomers, respectively, farther along the helical assembly; (**C**) End view of the ARC-1 pilus model containing 21 monomers (model length ~275 Å); (**D**) Cross section of the ARC-1 pilus model filament (~22 Å thick), in which residues from different monomers are shown in different colors. Aromatic rings of Phe1, Phe24 and Tyr27 are opaque and off-center in the cross section; (**E**) Cross section of the Geobacter homology model based on Pseudomonas; (**F**) Absence of a central channel in the ARC-1 model (left) vs. the central channel in the homology model (right).

The aromatic rings from the ARC-1 model have a compact geometry that may provide a potentially electrically conductive pathway (Fig. 4A). Three residues, Phe1, Phe24 and Tyr27 from different monomers stack with each other, and exhibit a helical distribution in the core of the filament (Fig. 4A,C,D). The off-center aromatic rings are close to the surface of the filament (Fig. 4D), which may explain why charge propagation could be observed with electrostatic force microscopy^12^. The Phe24 and Tyr27 from protomer P are closely aligned with Phe1 from protomers P + 3 and P + 4, and to form a continuous chain of aromatic rings (Fig. 4B). Distances between proximal carbon atoms in neighboring aromatic rings are 3.5 or 3.6 Å and very close to the reported stacking distance of ~3.5 Å^13^,^30^. The distances shown here are invariant through the length of the model, because all the monomers are identical in each model.

Separation between the centers of mass was taken into account in a previous study of pi-stacking^43^, and thus the center-to-center spacing is an appropriate alternative estimation of the distance between aromatic rings. Those values (4.1–5.5 Å) were comparable to previously reported distances^43^ (~5 Å), and the separation is consistent with metallic-like electron transport. The planes of the aromatic rings are not strictly parallel or perpendicular to each other and vary among the lowest energy models. However, strictly parallel alignment of aromatic residues is not a requirement for metallic-like conductivity^30^.

Unlike other models of the *G. sulfurreducens* pilus that have been based on homology modeling^13^,^16^, these lowest energy models, including the ARC-1, predict no central channel inside the filament (Fig. 4D,F). Our modeling revealed that the absence of a central channel brings the aromatics Phe1, Phe24 and Tyr27 closer together than that previously predicted with homology modeling (Fig. 4E,F), whose channel could be an artefact of the template^13^. Thus, the absence of a central channel might be essential for the high conductivity of the *G. sulfurreducens* pilus.

**Figure 5:**
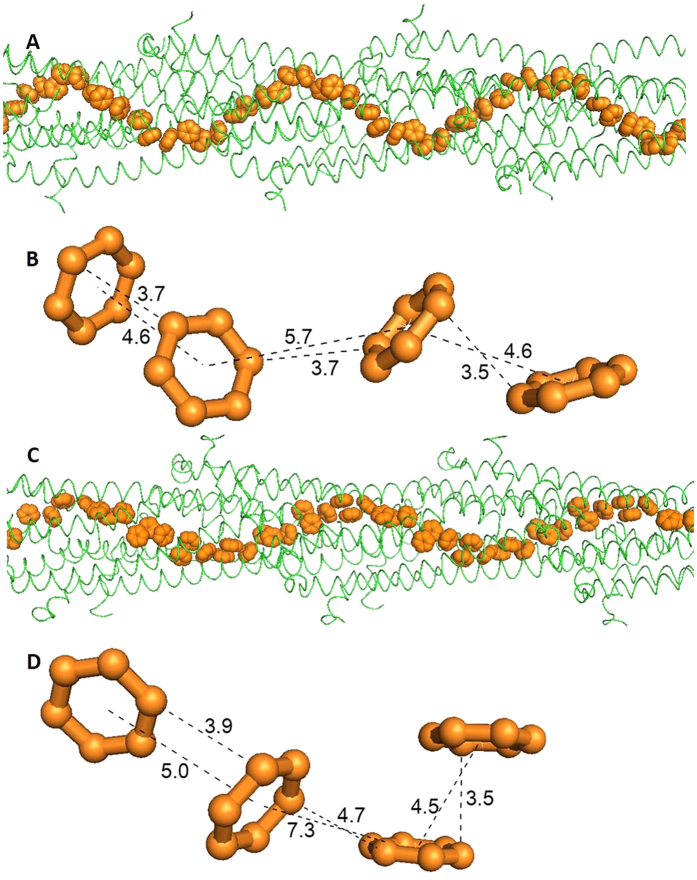
The alignment and details of aromatic rings from two other models among the 50 lowest-energy models. One exhibits a larger rotation angle (67.9°, **A** and **B**) than most, whereas the other shows a smaller angle (52.9°, **C** and **D**).

In addition to the ARC-1 model, about half of the 50 lowest-energy models have similar aromatic pathways in their cores, although the symmetry details vary. For example, another model has an azimuthal angle of 67.9°, an axial rise of 10.5Å, and a diameter of 36.1 Å (Fig. 5A,B). Although the rotational angle is larger than most models in the 50 lowest-energy group, corresponding to fewer subunits per turn (~5.3), the aromatic rings still align in a close pathway. Similarly, the Phe24 and Tyr27 from a reference protomer P are close to Phe1 from P + 3 and P + 4 (Fig. 5A,B). Another model, having a smaller rotation angle of 52.9°, has a similar chain of aromatic rings (Fig. 5C,D). All these models, with different details of assembly, predict the possibility of a continuous pathway of aromatic rings in the core of the *G. sulfurreducens* pili, and indicate that the first 3 aromatic residues, Phe1, Phe24 and Tyr27 along the N-terminal alpha-helices might be decisive in the electrical conductivity of the *G. sulfurreducens* pili.

### The constraints on aromatic residues were not required for convergence

**Figure 6:**
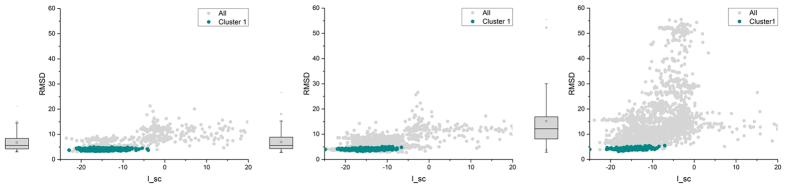
RMSD landscapes from the ARC-1 model versus energy scores. The grey plots and the boxes show the distributions of RMSDs for all the models from each calculation, and the dark cyan plots show the distribution of cluster 1 (the largest cluster). (**A**) Models with constraints on both charged and aromatic residues. (**B**) Models with constraints only on charged residues. (**C**) Models with no constraints.

As suggested by previous work of Type IV Pili modeling^17^, the use of proper constraints may drastically reduce the conformational search space without changing the tendency towards convergence. The fixed-backbone docking with rotation angles between 40° and 60° was repeated for three different combinations of constraints: (i) constraints on both charged and aromatic residues; (ii) constraints only on charged residues and (iii) no constraint. The RMSDs from the ARC-1 structure have also been calculated for all the models. The results (Fig. 6) show that the biggest cluster (shown as dark cyan plots) is always near the ARC-1 model no matter which group of constraints was employed, and the models always tend to converge near the largest cluster, even when there were no constraints. Meanwhile, models with constraints on both charged and aromatic residues (Fig. 6A) are slightly more convergent than the models with only constraints on charged residues (Fig. 6B). Both groups of models seem much more convergent than the group without constraint (Fig. 6C). Thus, the constraints employed for the docking were not required for convergence, and the models with closely packed aromatics are more likely reasonable structures rather than artefacts.

### Salt bridges and cation-pi interactions stabilizing the pili structures

**Figure 7:**
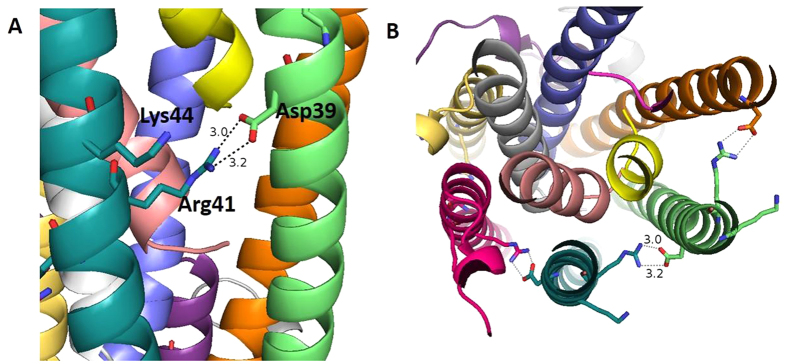
Salt bridge in the ARC-1 pilus model formed by Arg41 and Asp39 may contribute to the stabilization of the pilus; (**B**) Cross section showing the salt bridge.

Charged residue pairs and cation-pi interactions can contribute to the assembly and stabilization of pili^18^,^21^ and thus were analyzed. Charged residue pairs were counted with a Perl script for the top 50 models. 80% of these models have a salt bridge between Arg41 and Asp39, which means the distances between the oxygen atoms of acid side chains and the nitrogen atoms of basic side chains are less than 4 Å^44^. For example, in the ARC-1 model, the Arg41 from one monomer and the Asp39 from the neighboring monomer are close to each other; the distance between the nitrogen and oxygen atoms is ~3 Å (Fig. 7A). These predicted salt bridges might contribute to the stabilization of the pili, as they form a continuous interaction chain between each pair of neighboring subunits (Fig. 7B). Moreover, the residues Arg41 and Lys44 are aligned on the same side of the alpha-helix, which may form a positive patch on the surface of monomers and then interact with the negative charged area on the filament, as a driving force during the assembly process of pili.

Cation-pi interactions were determined for the 50 lowest-energy models with the CaPTURE server^45^,^46^. 28% of the 50 models have a cation-pi interaction between Phe24 and Arg28, within the same subunit, deemed energetically significant by the CaPTURE server. This interaction involves Phe24, which is part of the proposed electrically conductive pathway and may affect the electrical properties of the pilus. These features, including both the salt bridges and cation-pi interactions, could be evaluated by charge reversal experiments^19^,^21^, and thus provide an approach for future testing of these models.

## Conclusions

These results demonstrate that it is feasible for the pilin monomers of *G. sulfurreducens* to assemble into a highly stable filamentous structure in which a core chain of aromatic amino acids facilitate electron transport along the length of the pilus. This finding is consistent with multiple lines of experimental evidence that have consistently suggested that *G. sulfurreducens* pili have metallic-like conductivity^3^,^10^,^12^. No model can prove or disprove experimental results, but it is necessary for a model to be consistent with experimental observations in order to provide a framework to summarize what is known about a system and to guide further investigation.

Ultimately, a more definitive determination of the *G. sulfurreducens* pilus structure with techniques such as cryo-EM^18^ is desirable, but the very small diameter (3 nm) of the *G. sulfurreducens* pilus will make this already technically challenging approach even more difficult. In the meantime, the models presented here provide the basis for hypotheses that can be further experimentally evaluated to better understand the novel mode of biological electron transport along *Geobacter* pili.

## Additional Information

**Data Availability**: Animations and atomic coordinates (in PDB format) of the models described in detail here can be downloaded from Proteopedia.Org/w/Ke_Xiao/1. Interactive 3D views of GS pilus filament models described here are available at Proteopedia.Org/w/Ke_Xiao/1.

**How to cite this article**: Xiao, K. *et al*. Low Energy Atomic Models Suggesting a Pilus Structure that could Account for Electrical Conductivity of *Geobacter sulfurreducens* Pili. *Sci. Rep.* **6**, 23385; doi: 10.1038/srep23385 (2016).

## Supplementary Material

Supplementary Information
