# Supplementary material for: Low Energy Atomic Models Suggesting a Pilus Structure that could Account for Electrical Conductivity of Geobacter sulfurreducens Pili
Source: Sci Rep. 2016 Mar 22;6:23385. doi: 10.1038/srep23385 (PMC4802205; doi:10.1038/srep23385)
Supplement: Supplementary Information [file srep23385-s1.pdf]

Low Energy Atomic Models Suggesting a Pilus Structure that could Account for Electrical Conductivity of *Geobacter sulfurreducens* Pili

Ke Xiao[1, 2], Nikhil S. Malvankar[2, 3], Chuanjun Shu[1], Eric Martz[2], Derek R. Lovley[2] and Xiao Sun[1*]

[1]State Key Laboratory of Bioelectronics, School of Biological Science and Medical Engineering, Southeast University, Nanjing, 210096, China. E-mail: xsun@seu.edu.cn

[2]Department of Microbiology, University of Massachusetts, Amherst, Massachusetts, USA

[3]Present address: Department of Molecular Biophysics and Biochemistry, Microbial Sciences Institute, Yale University.

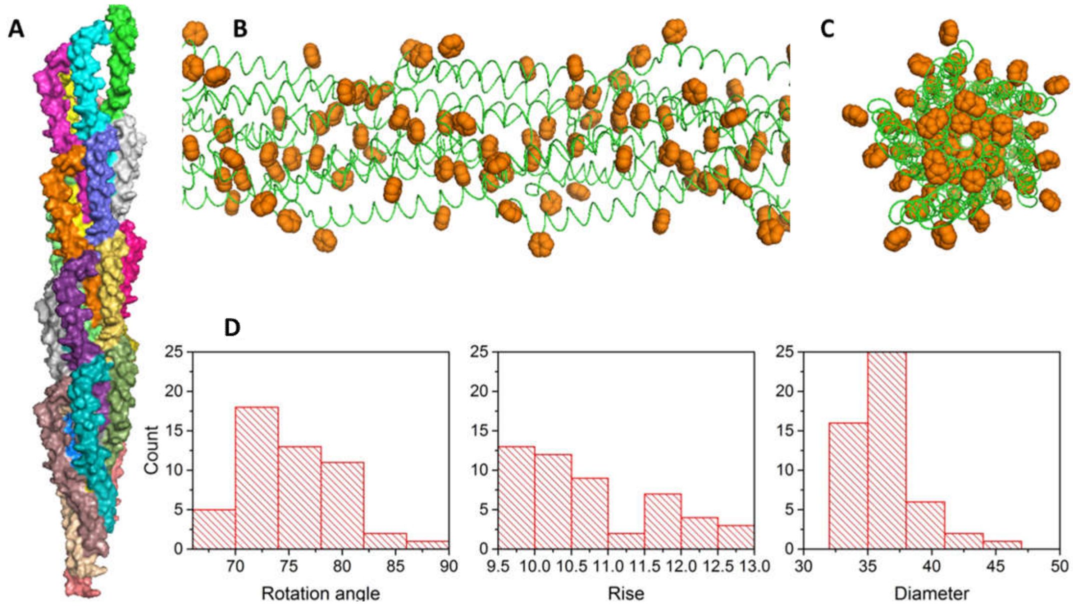

Figure S1. Models from the 60-80˚ group. A. A selected model from this group containing 21 subunits, each a different color; B. Overview of the aromatic rings in the model. The aromatic rings can cluster into small groups, but the distances between different groups appear to be too long for electron transfer; C. End view of the model containing 21 monomers; D. Overview of the symmetry features of the 50 lowest-energy models from this group. The symmetry parameters seem scattered (less convergent), when compared to the 40-60˚ and 100-120˚ groups.

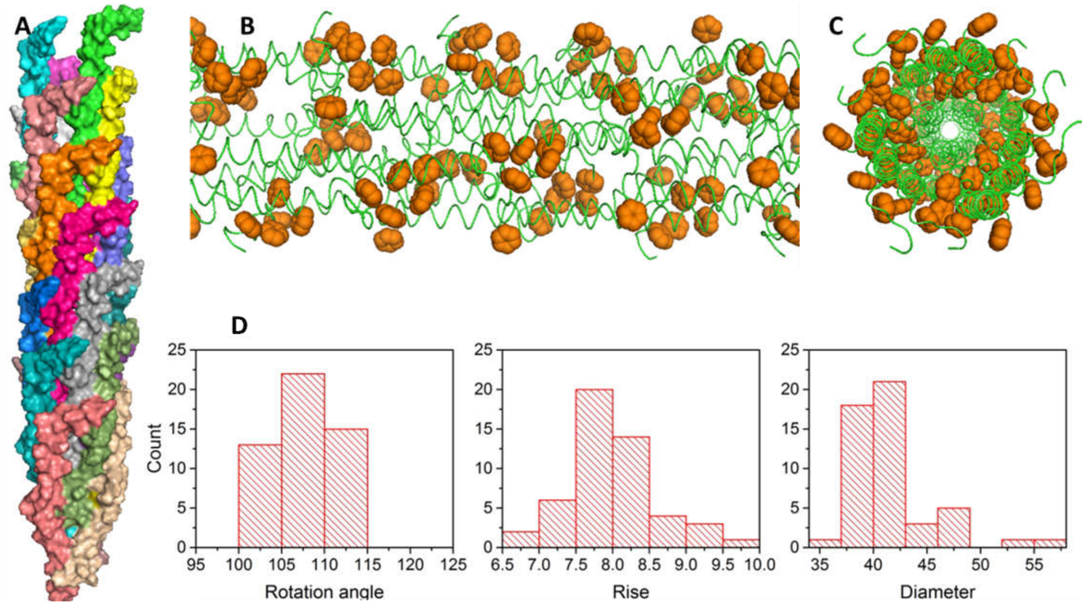

Figure S2. Models from the 100-120˚ group. A. A selected model from this group containing 21 subunits, each a different color; B. Overview of the aromatic rings in the model. The distances between different aromatic groups appear to be too long for electron transfer; C. End view of the model containing 21 monomers; D. Overview of the symmetry features of the 50 lowest-energy models from this group. The rotation angles are near 107 degrees, corresponding to ~3.4 subunits per turn. Most models have an axial rise between 7.5 and 8.5 Å, and the diameters of models are near 40 Å.

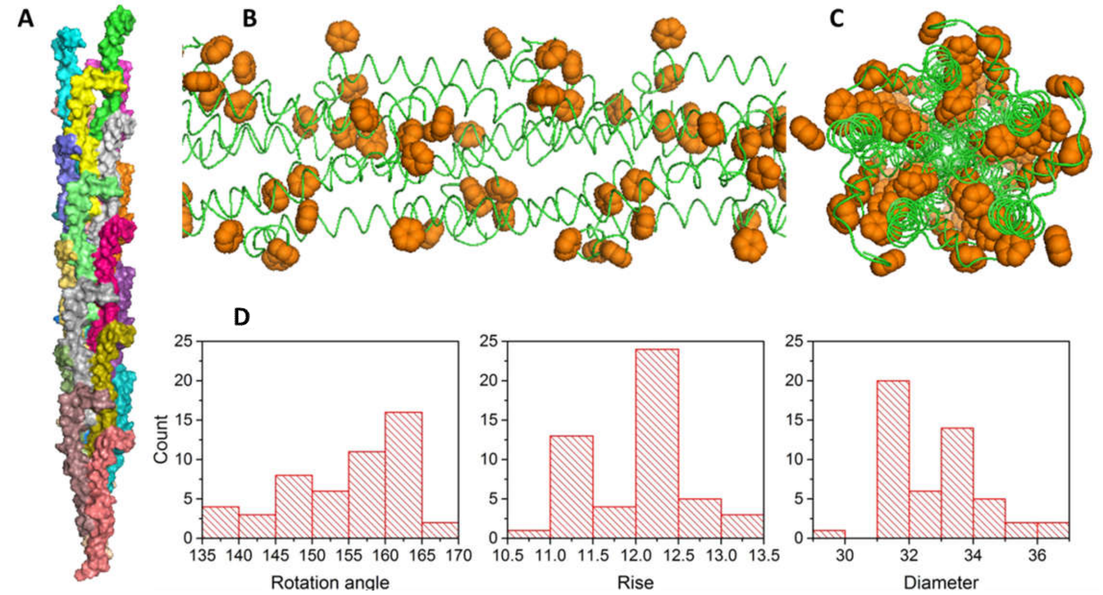

Figure S3. Models from the 130-160˚ group. A. A selected model from this group containing 21 subunits, each a different color; B. Overview of the aromatic rings in the model, which are not close enough to form a continuous pathway; C. End view of the model containing 21 monomers; D. Overview of the symmetry features of the 50 lowest-energy models from this group, which are scattered in a wider range (less convergent), when compared to the 40-60˚ and 100-120˚ groups.

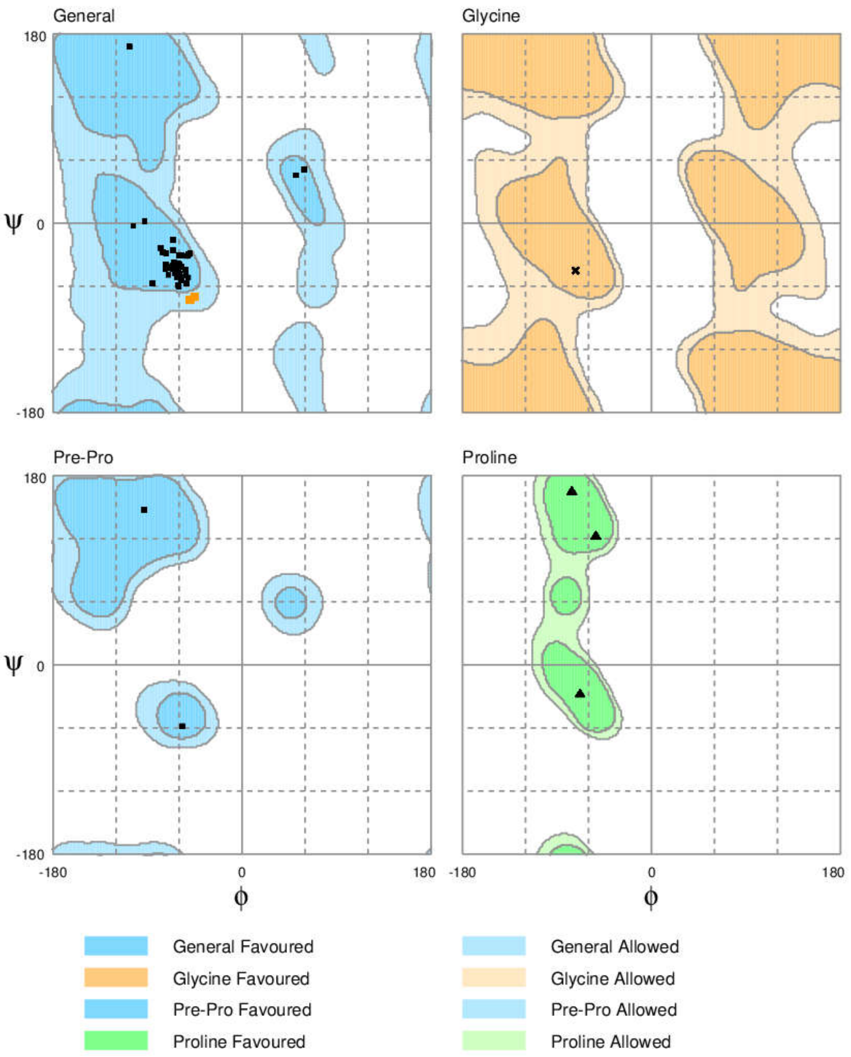

Figure S4. Ramachandran plots of an example structure from the final models. Number of residues in favored region (~98% expected): 1197 (96.6%). Number of residues in allowed region (~2.0% expected): 42 (3.4%). Number of residues in disallowed region: 0 (0.0%)

Animations, interactive views, and downloadable models

Animations and atomic coordinates (in PDB format) of the models described in detail here can be downloaded from Proteopedia.Org/w/Ke_Xiao/1.

Interactive 3D views of GS pilus filament models described here are available at Proteopedia.Org/w/Ke_Xiao/1.
